# Supplementary material for: N6-methyladenosine RNA modification regulates strawberry fruit ripening in an ABA-dependent manner
Source: Genome Biol. 2021 Jun 3;22:168. doi: 10.1186/s13059-021-02385-0 (PMC8173835; doi:10.1186/s13059-021-02385-0)
Supplement: Supplementary file 1 — Additional file 1: Figure S1. Pearson correlation analysis of input reads in the m6A peak regions identified from m6A-seq. Figure S2. Pearson correlation analysis of immunoprecipitation (IP) reads in the m6A peak regions identified from m6A-seq. Figure S3. Validation of confident m6A peaks. Figure S4. Sequence motif identified within the m6A peaks by HOMER (http://homer.ucsd.edu/homer/). Figure S5. LC-MS/MS assay revealing the total m6A levels in strawberry fruit at S6, RS1, and RS3 stages. Figure S6. Heat map of m6A enrichment ratios of differential m6A peaks identified in fruit at RS1 stage compared to those at S6 stage. Figure S7. Gene Ontology (GO) analysis of transcripts containing m6A peaks during strawberry ripening. Figure S8. The m6A modification and expression level for gene in ABA pathway and gene encoding translation initiation factors and elongation factors in octoploid cultivated strawberry. Figure S9. Validations of specific m6A sites in the NCED5, ABAR, and AREB1 transcripts. Figure S10. Identification of the conserved MT-A70 domain of m6A methyltransferases MTA and MTB in diploid woodland strawberry and octoploid cultivated strawberry. Figure S11. Protein sequence alignment of the MT-A70 domains in m6A methyltransferases. Figure S12. Influence of MTA silencing on mRNA stability. Figure S13. The changes in translation efficiency of ABA signaling gene and ripening genes in the MTA-silenced or the MTA-overexpressed fruits. Figure S14. Informations of m6A-seq in the MTA-silenced fruits and the control. Figure S15. m6A enrichment and gene expression for the SlDML2 homologs in strawberry fruit. Figure S16. m6A enrichment for DNA methyltransferase genes from m6A-seq data. Figure S17. Regulatory model for the m6A-mediated ripening in climacteric tomato fruit and non-climacteric strawberry fruit. [file 13059_2021_2385_MOESM1_ESM.pdf]

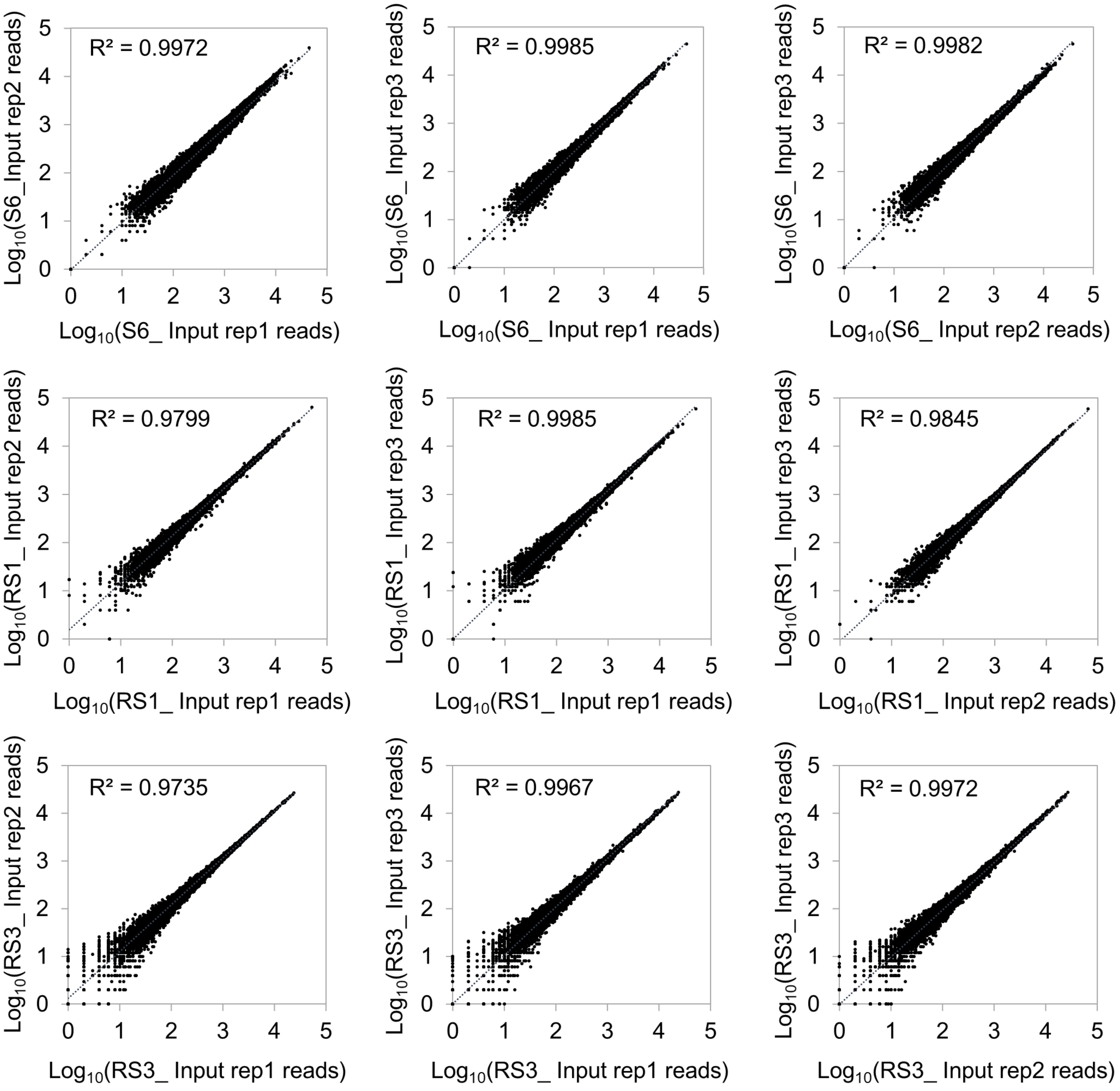

Additional file 1: Figure S1. Pearson correlation analysis of input reads in the m^6A peak regions identified from m^6A-seq. m^6A-seq was performed with three independent biological replicates in strawberry fruit at S6, RS1, and RS3 stages, respectively. S6, the growth stage 6; RS1, the ripening stage 1; RS3, the ripening stage 3; R, Pearson correlation coefficient; Rep, Replicate.

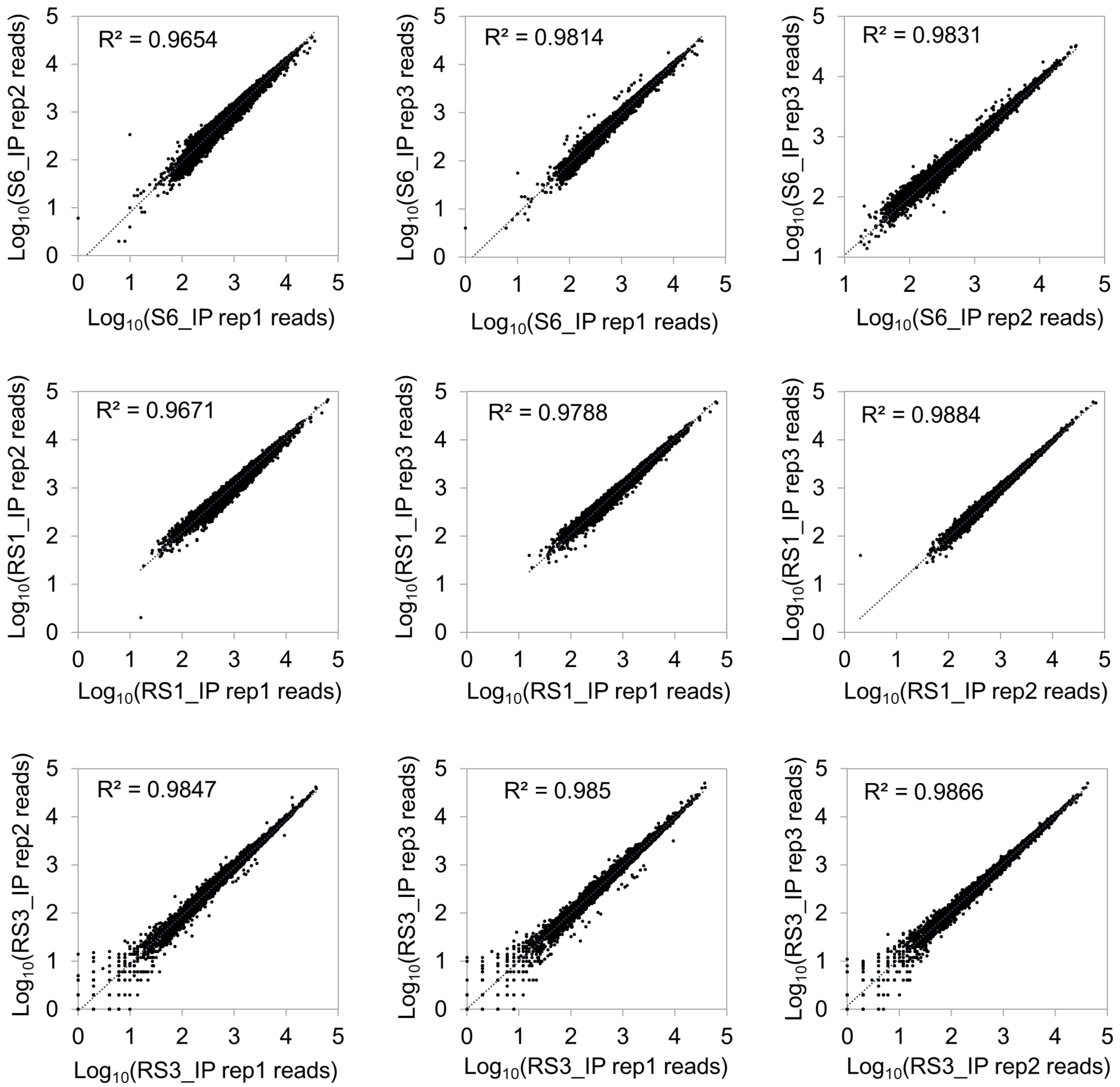

Additional file 1: Figure S2. Pearson correlation analysis of immunoprecipitation (IP) reads in the m^6A peak regions identified from m^6A-seq. m^6A-seq was performed with three independent biological replicates in strawberry fruit at S6, RS1, and RS3 stages, respectively. S6, the growth stage 6; RS1, the ripening stage 1; RS3, the ripening stage 3; R, Pearson correlation coefficient; Rep, Replicate.

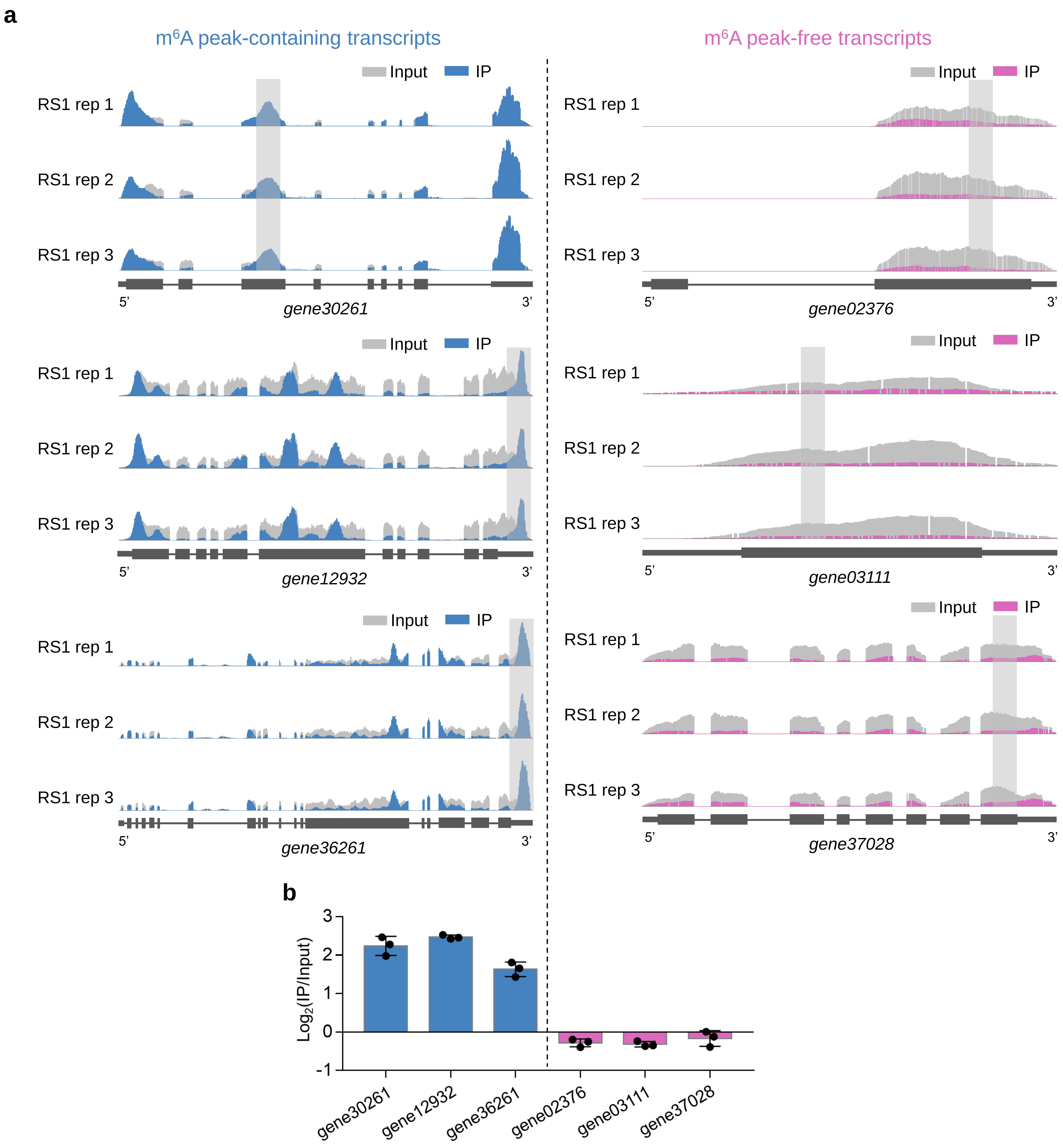

Additional file 1: Figure S3. Validation of confident m⁶A peaks. a Integrated Genome Browser (IGB) tracks showing the distribution of m⁶A reads in three m⁶A peak-containing transcripts (*gene30621*, *gene12932*, and *gene36261*) and three m⁶A peak-free transcripts (*gene02376*, *gene03111*, and *gene37028*) in strawberry fruit at RS1 stage. RS1, the ripening stage 1. **b** Validations of the m⁶A enrichment by m⁶A-immunoprecipitation (IP)-qPCR in the m⁶A peak regions, as well as the negative control regions, indicated by the shadow boxes in **a**. Data are presented as mean ± standard deviation (n = 3).

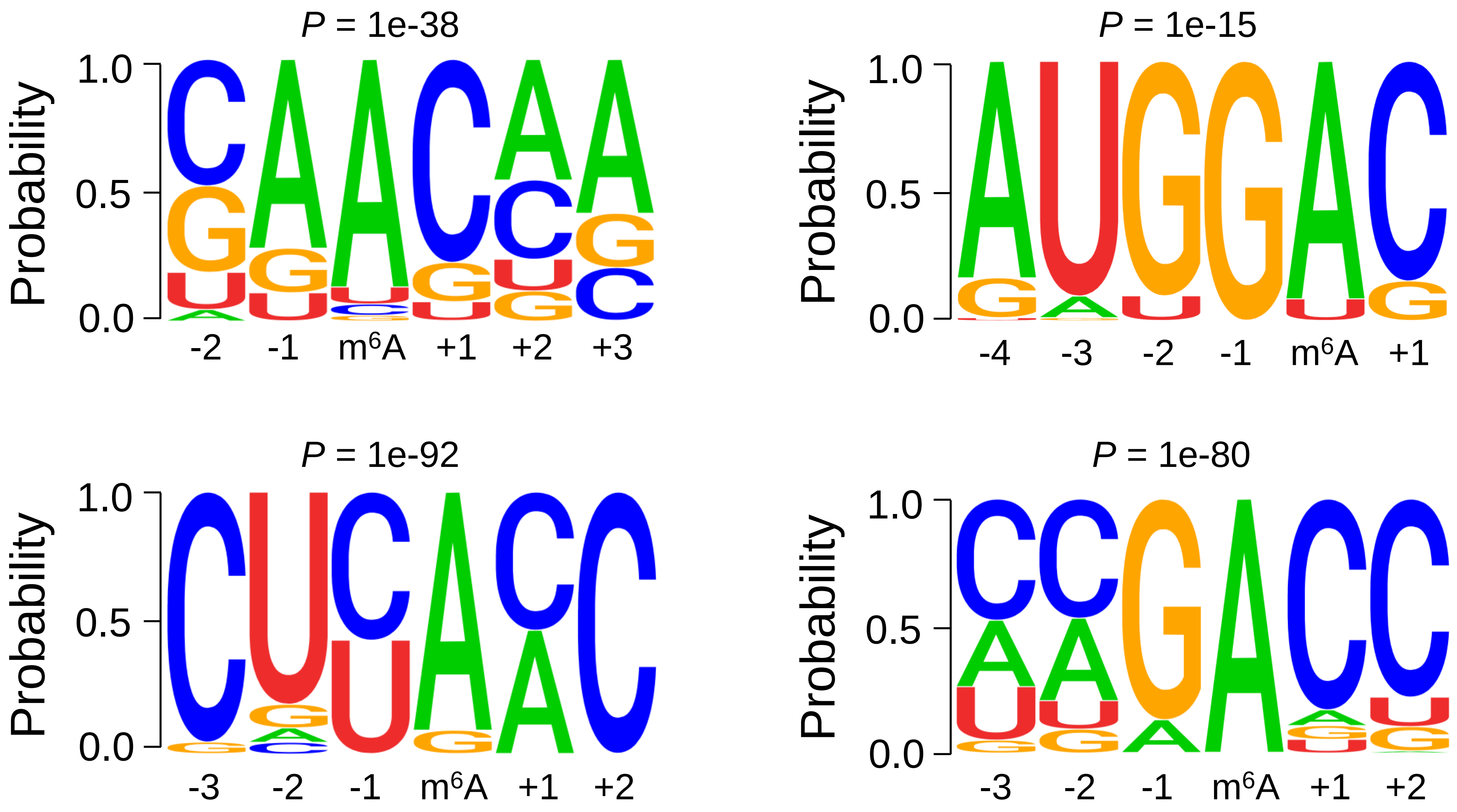

Additional file 1: Figure S4. Sequence motif identified within the m⁶A peaks by HOMER (http://homer.ucsd.edu/homer/).

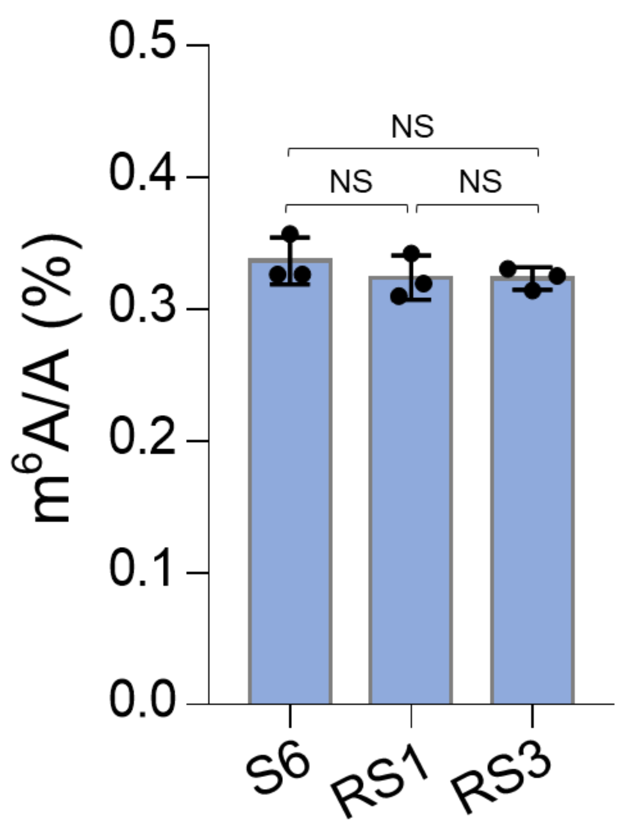

Additional file 1: Figure S5. LC-MS/MS assay revealing the total m⁶A levels in strawberry fruit at S6, RS1, and RS3 stages. Data are presented as mean ± standard deviation (n = 3). S6, the growth stage 6; RS1, the ripening stage 1; RS3, the ripening stage 3; NS, no significance.

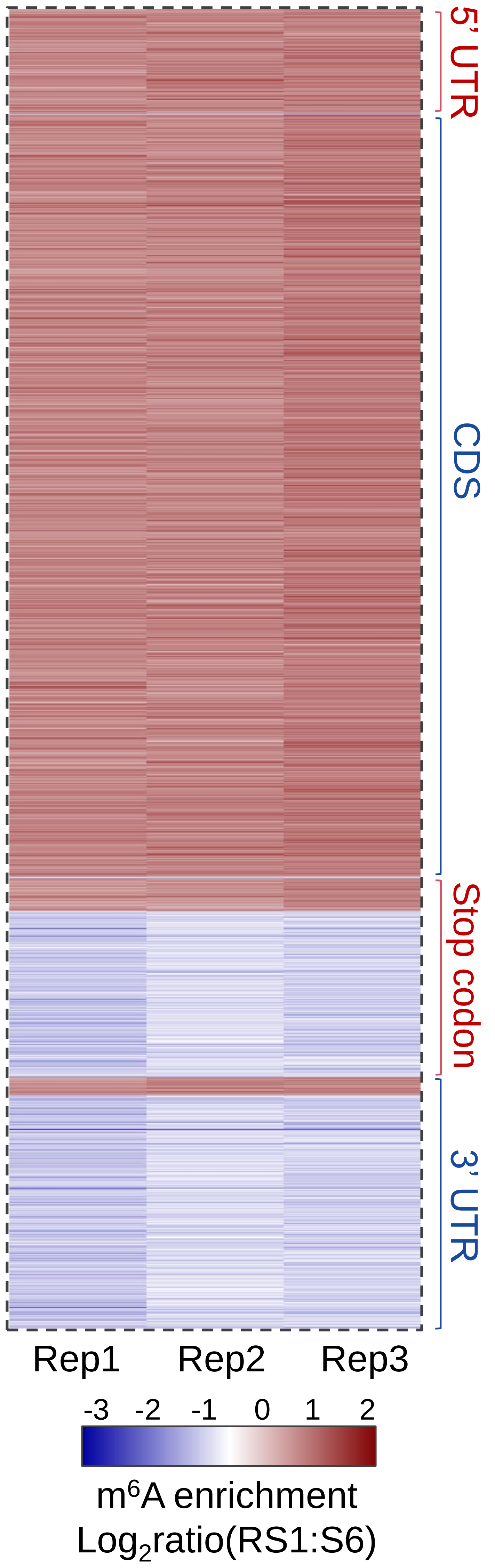

Additional file 1: Figure S6. Heat map of m^{6}A enrichment ratios of differential m^{6}A peaks identified in fruit at RS1 stage compared to those at S6 stage. All differential m^{6}A peaks were analyzed based on the m^{6}A distributions. S6, the growth stage 6; RS1, the ripening stage 1; Rep, replicate.

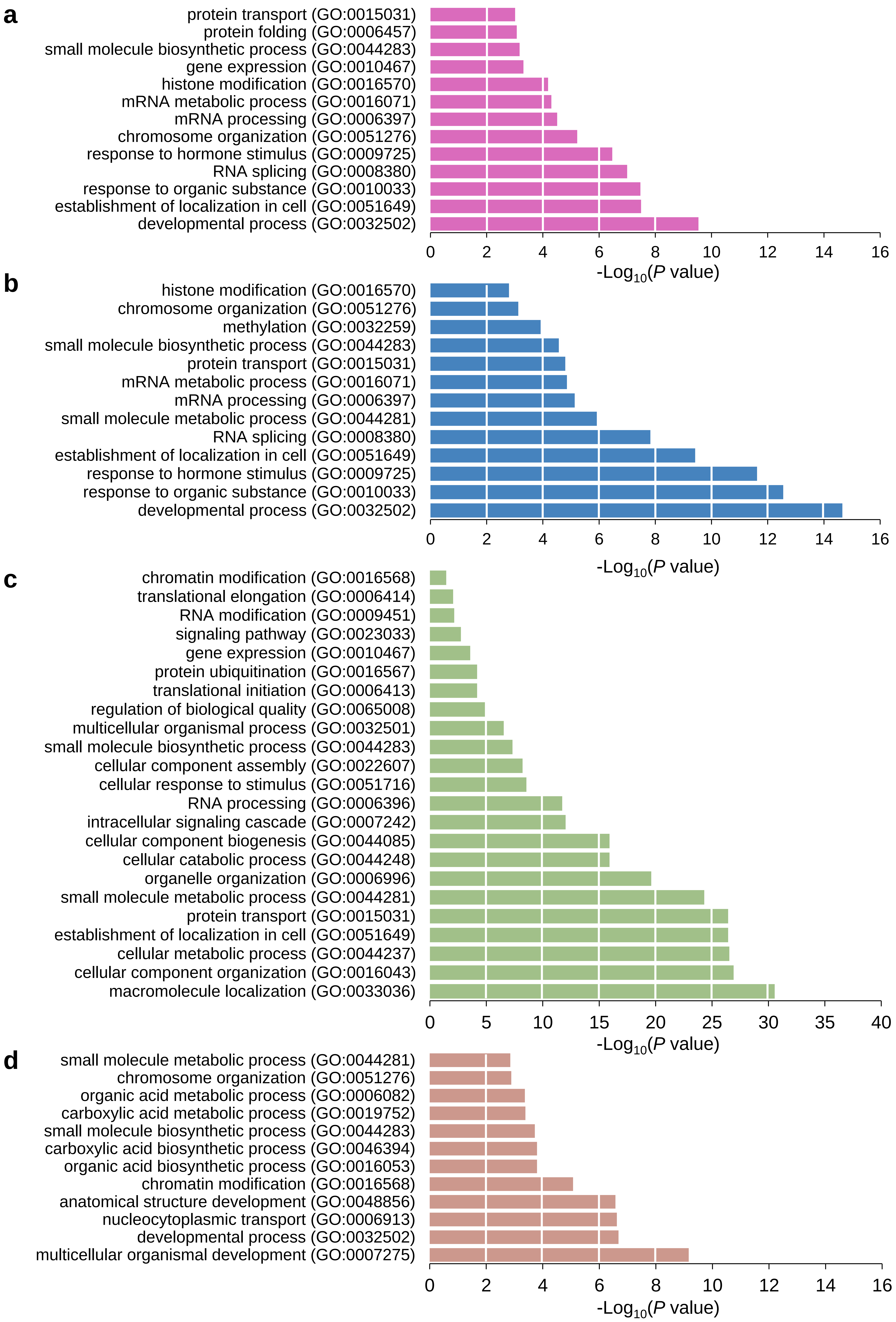

Additional file 1: Figure S7. Gene Ontology (GO) analysis of transcripts containing m⁶A peaks during strawberry ripening. a GO enrichment for the transcripts containing ripening-specific m⁶A peaks. **b** GO enrichment for the transcripts containing differential m⁶A peaks in fruit at RS1 stage compared to those at S6 stage. **c** GO enrichment for the transcripts containing non-differential m⁶A peaks in fruit at RS1 stage compared to those at S6 stage. **d** GO enrichment for the ripening-induced and ripening-repressed transcripts containing differential m⁶A peaks in fruit at RS1 stage compared to those at S6 stage. The GO enrichment was analyzed on the agriGO database (http://systemsbiology.cau.edu.cn/agriGOv2/), and the most impacted GO biological process terms with a statistical significance of Yekutieli-corrected *P* value < 0.05 were shown.

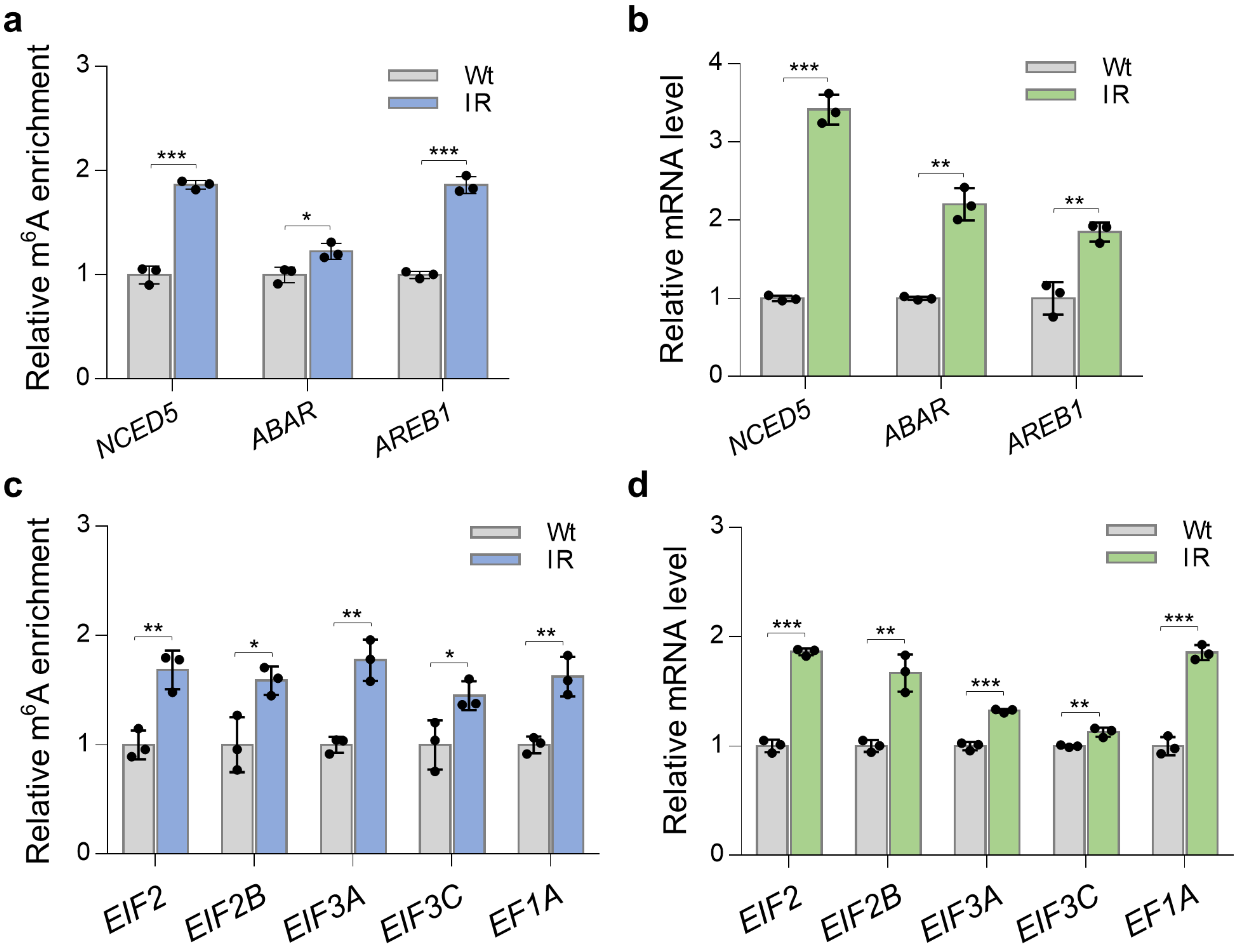

Additional file 1: Figure S8. The m^{6}A modification and expression level for gene in ABA pathway and gene encoding translation initiation factors and elongation factors in octoploid cultivated strawberry. The relative m^{6}A enrichment and gene expression for gene in ABA pathway (**a** and **b**) and gene encoding translation initiation factors and elongation factors (**c** and **d**) were determined by m^{6}A-IP-qPCR and quantitative RT-PCR, respectively. The *ACTIN* gene served as an internal control. Data are presented as mean ± standard deviation (n = 3). Asterisks indicate significant differences (**P* < 0.01, ***P* < 0.01, ****P* < 0.001; Student's t test). Wt, white; IR, initial red.

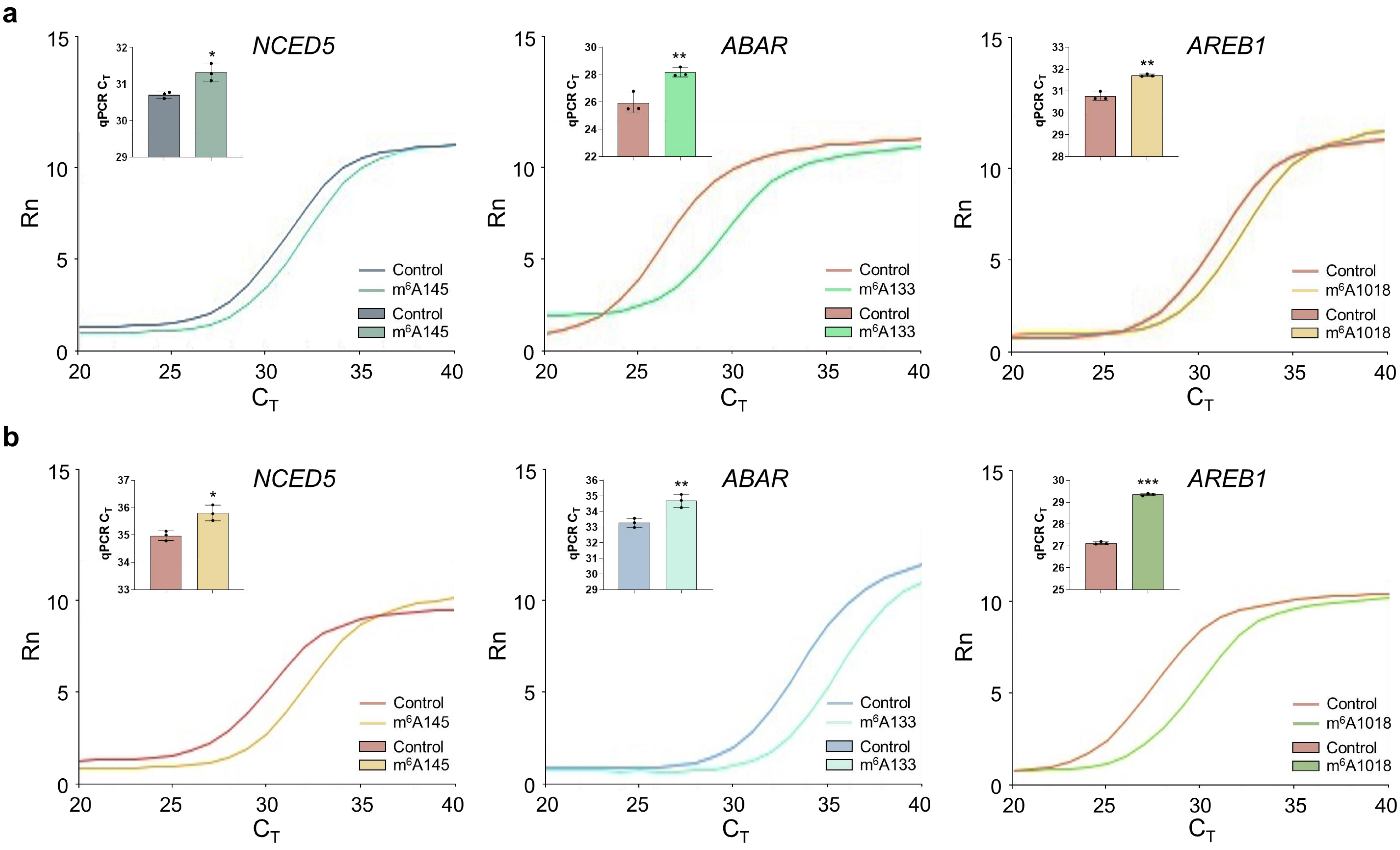

Additional file 1: Figure S9. Validations of specific m^{6}A sites in the *NCED5*, *ABAR*, and *AREB1* transcripts. a and **b** Representative real-time fluorescence amplification curves and bar plots of the threshold cycle (C_T) of qPCR for detecting m^{6}A145 in *NCED5*, m^{6}A133 in *ABAR*, and m^{6}A1018 in *AREB1* in strawberry fruit at RS1 stage (**a**) and *Nicotiana benthamiana* leaves (**b**). The non-adenosine sites adjacent to the specific m^{6}A site were used as the internal controls. Rn represents the raw fluorescence for the associated well that was normalized to the fluorescence of the passive reference dye (ROX). Data are presented as mean ± standard deviation (n = 3). Asterisks indicate significant differences (*P < 0.05, **P < 0.01, ***P < 0.001; Student's t test).

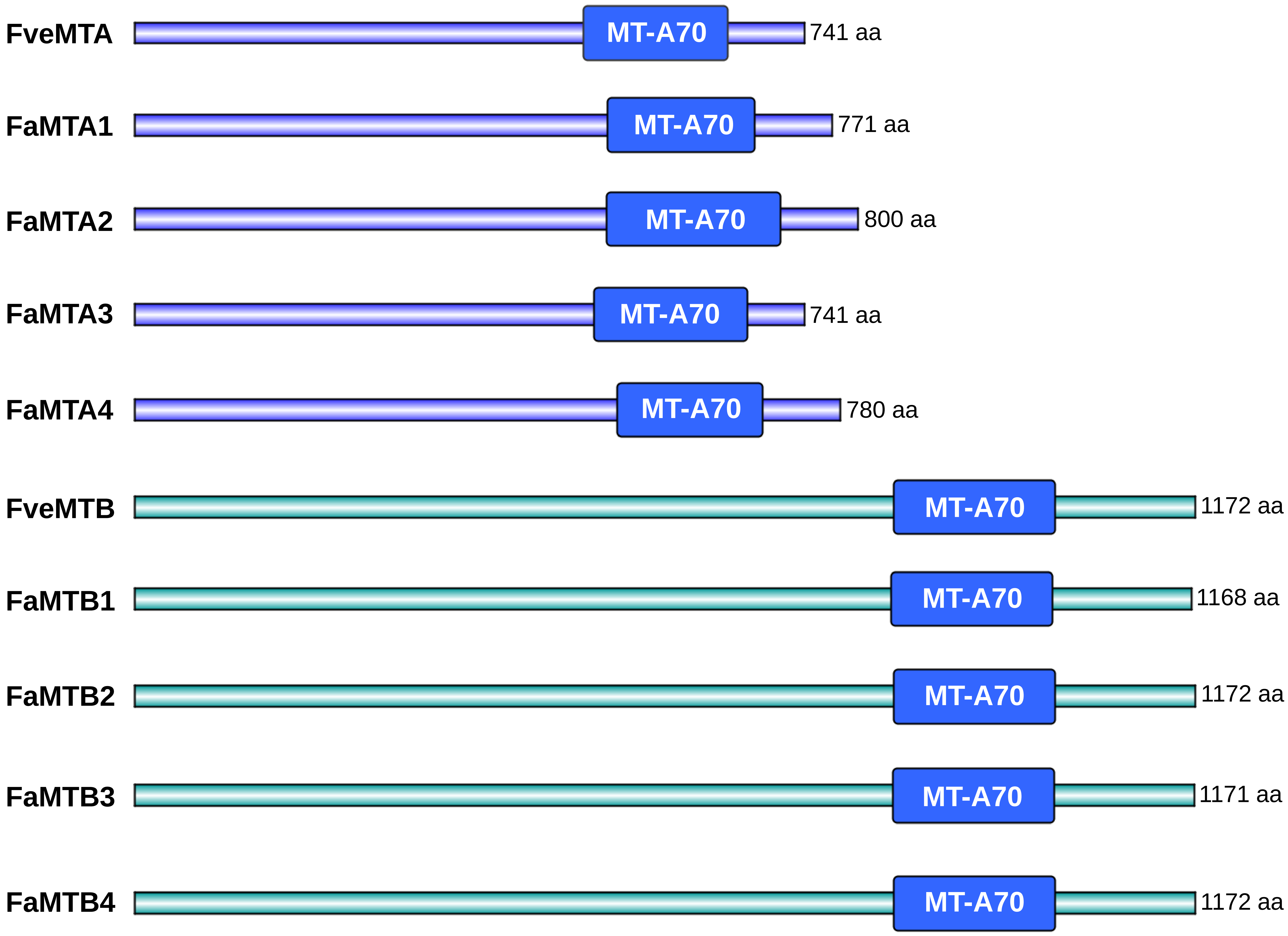

Additional file 1: Figure S10. Identification of the conserved MT-A70 domain of m⁶A methyltransferases MTA and MTB in diploid woodland strawberry and octoploid cultivated strawberry. Fve, *Fragaria vesca*; Fa, *Fragaria* × *ananassa*. The full-length protein sequences were analyzed by the online CDD software (https://www.ncbi.nlm.nih.gov/Structure/cdd/ wrpsb.cgi). aa, amino acid.

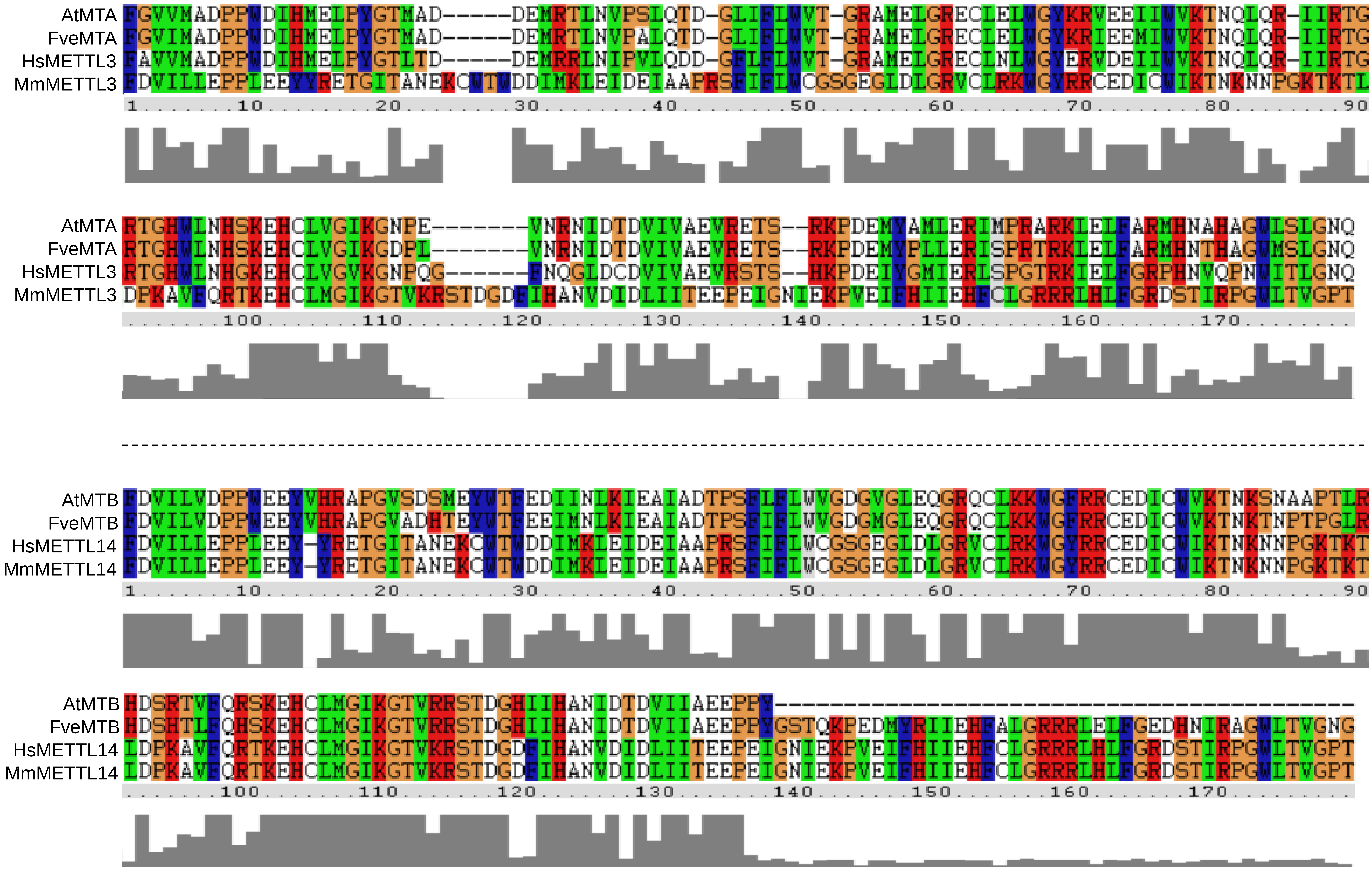

Additional file 1: Figure S11. Protein sequence alignment of the MT-A70 domains in m⁶A methyltransferases. The alignment was performed by using Clustal X (http://evomics.org/resources/software/bioinformatics-software/clustal-x/). Fve, *Fragaria vesca*; At, *Arabidopsis thaliana*; Hs, *Homo sapiens*; Mm, *Mus musculus*.

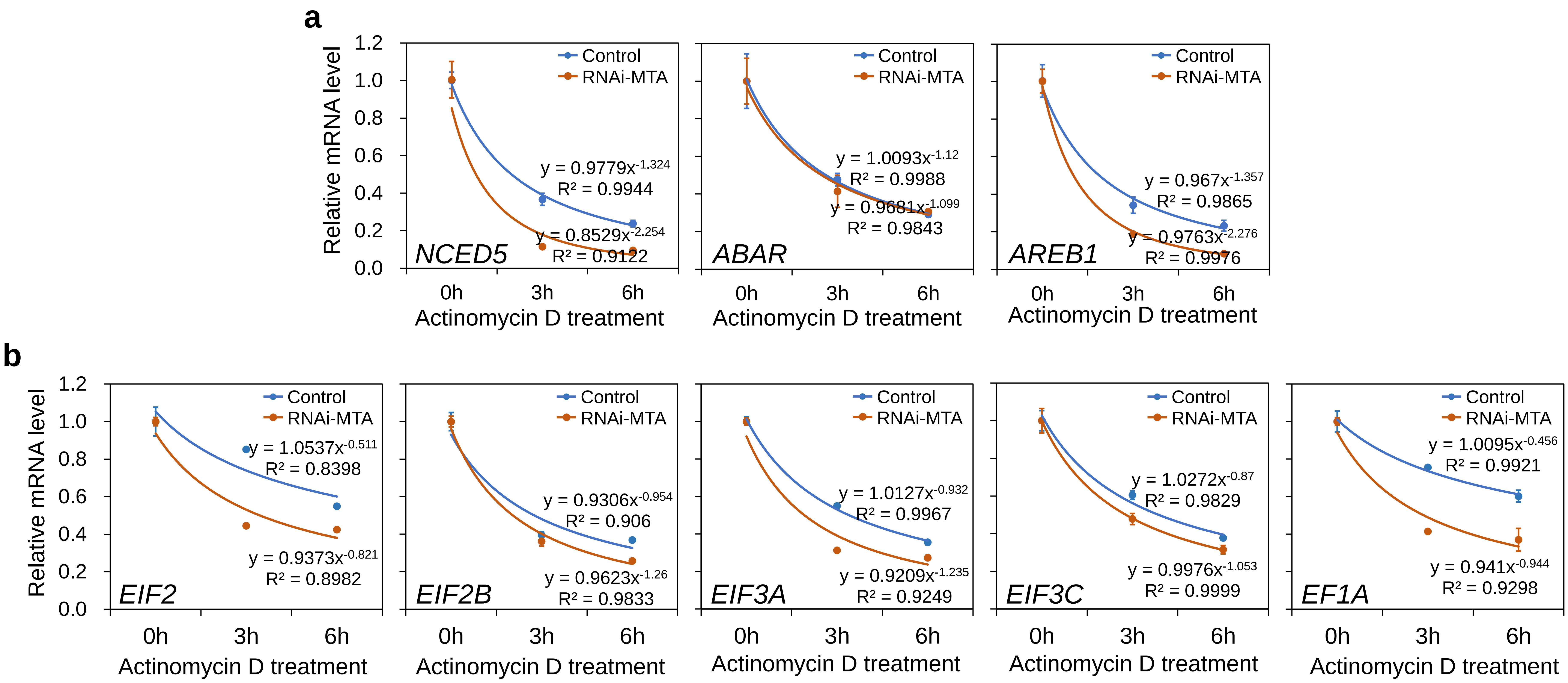

Additional file 1: Figure S12. Influence of *MTA* silencing on mRNA stability. The mRNA stability for gene in ABA pathway (**a**) and genes encoding translation initiation factors and elongation factors (**b**) were determined in the *MTA* RNAi fruits (RNAi-MTA) and the controls. The total RNAs were extracted after actinomycin D treatment at an indicated time point and submitted to quantitative RT-PCR assay. The *ACTIN* gene served as an internal control. Data are presented as mean ± standard deviation (n = 3).

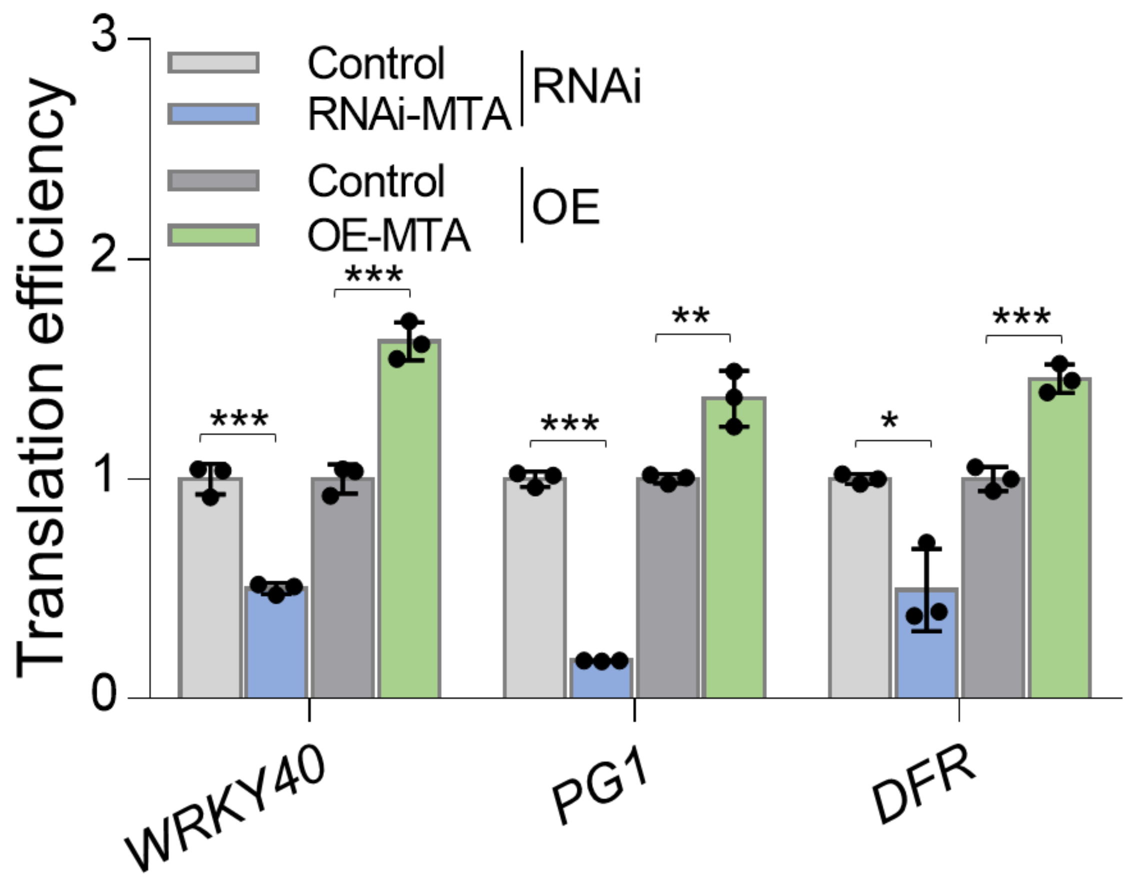

Additional file 1: Figure S13. The changes in translation efficiency of ABA signaling gene and ripening genes in the *MTA*-silenced or the *MTA*-overexpressed fruits. Translation efficiency was expressed as the abundance ratio of mRNA in the polysomal RNA versus the total RNA. Data are presented as mean ± standard deviation (n = 3). Asterisks indicate significant differences (**P < 0.01, ***P < 0.001; Student's t test). RNAi-MTA, *MTA* silencing; OE-MTA, *MTA* overexpression.

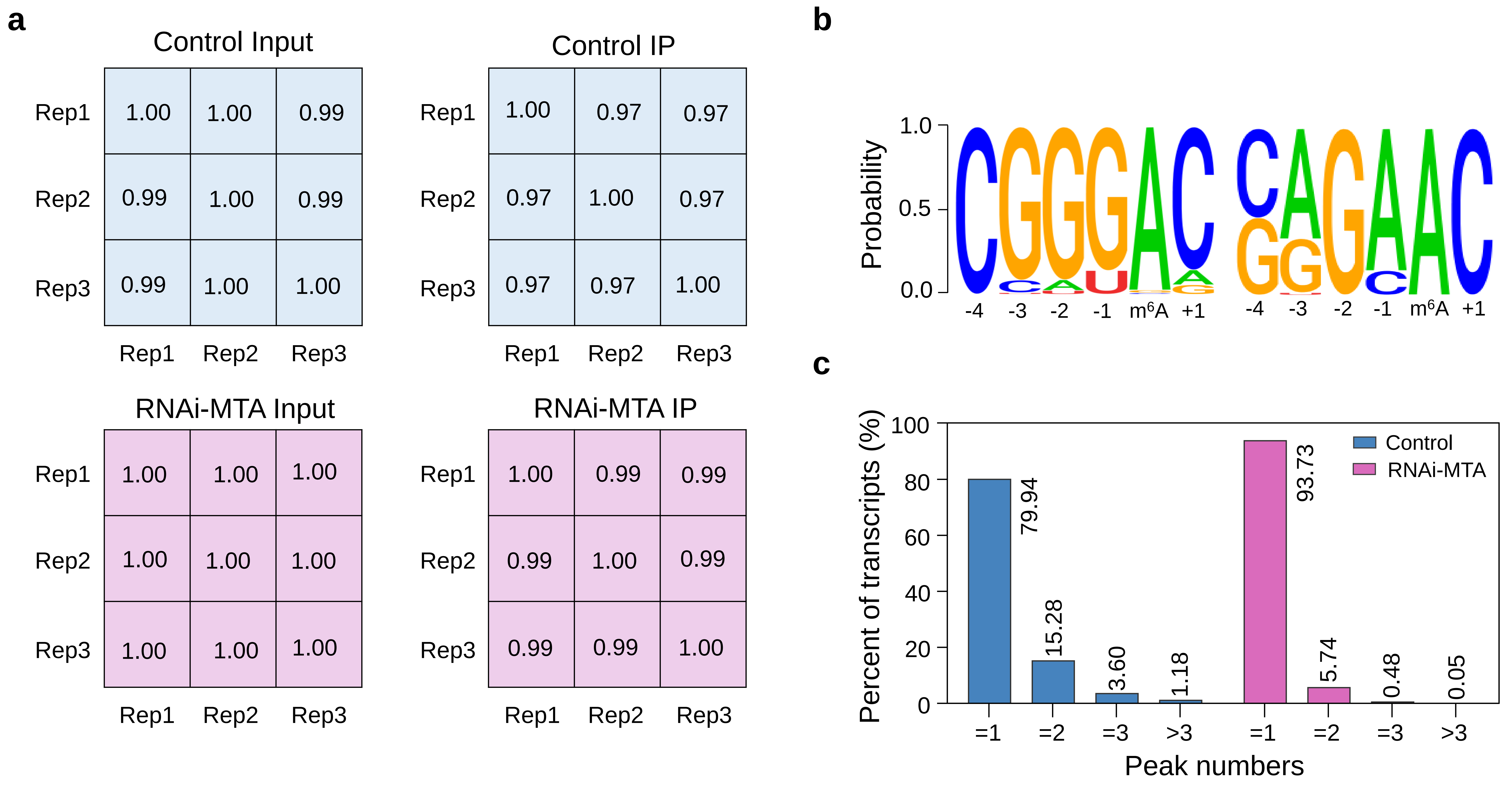

Additional file 1: Figure S14. Informations of m⁶A-seq in the *MTA*-silenced fruits and the controls. a Pearson correlation analysis of input and immunoprecipitation (IP) reads of the m⁶A peak regions identified from m⁶A-seq in the *MTA*-silenced (RNAi-MTA) fruits and the controls. Rep, replicate. **b** Sequence motif identified within the m⁶A peaks by HOMER (http://homer.ucsd.edu/homer/). **c** Percentage of the m⁶A-containing transcripts containing various m⁶A peak numbers.

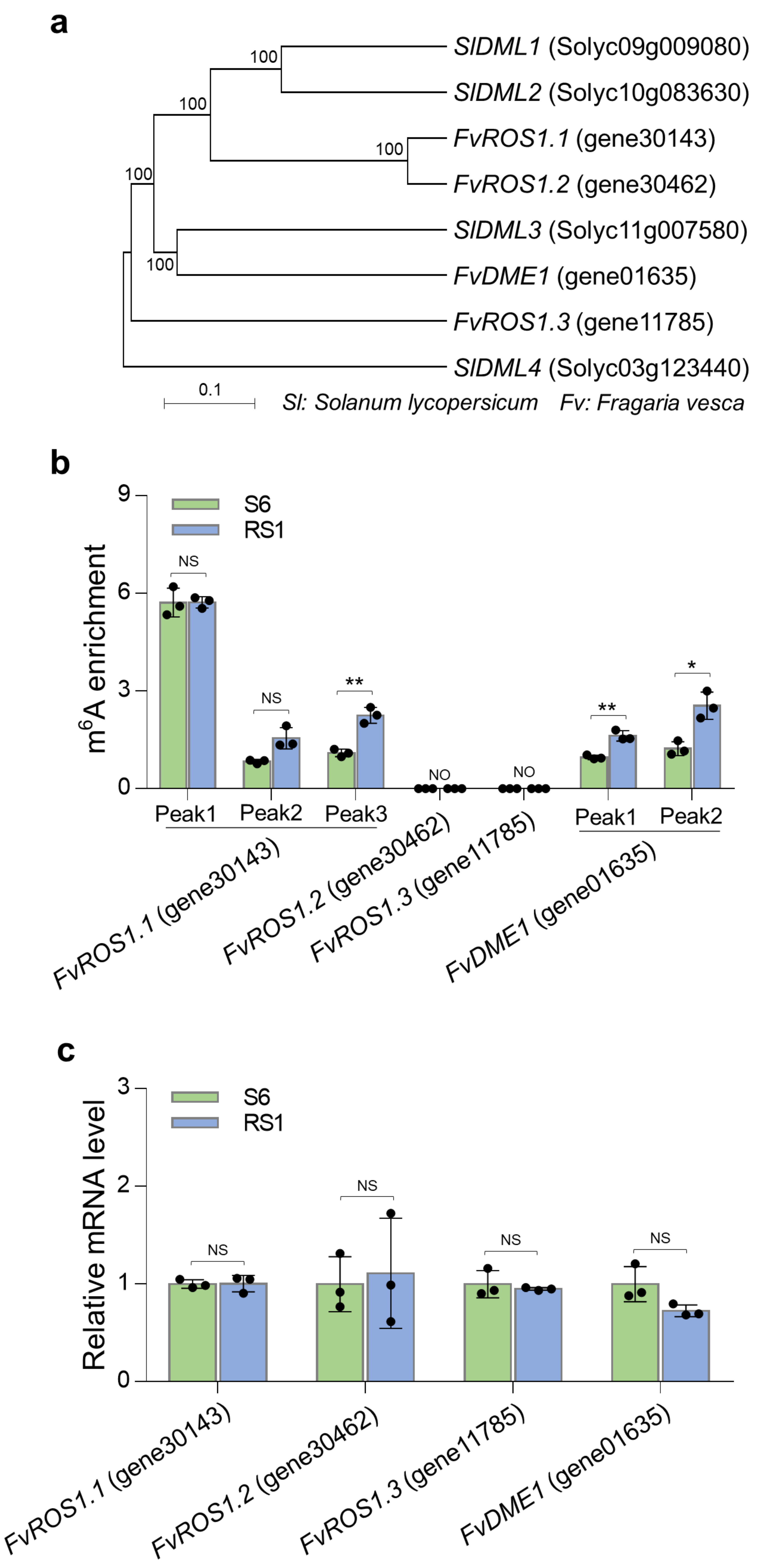

Additional file 1: Figure S15. m⁶A enrichment and gene expression for the *SlDML2* homologs in strawberry fruit. a Phylogenetic analysis of tomato DEMETER-like DNA demethylase (DML) genes and their homologs in strawberry. The phylogenetic tree was generated by MEGA (version 5.2). Bootstrap values from 1000 replications for each branch are presented. **b** m^6A enrichment for the *DML* homologs in strawberry fruit at S6 and RS1 stages as revealed by m^6A-seq. **c** Gene expression for the *DML* homologs in strawberry fruit at S6 and RS1 stages as revealed by quantitative RT-PCR analysis. The *ACTIN* gene served as an internal control. Data are presented as mean ± standard deviation (n = 3). Asterisks indicate significant differences (**P* < 0.05, ***P* < 0.01; Student's t test). 'NO' represents no confident m^6A peaks identified by m^6A-seq. NS, no significance.

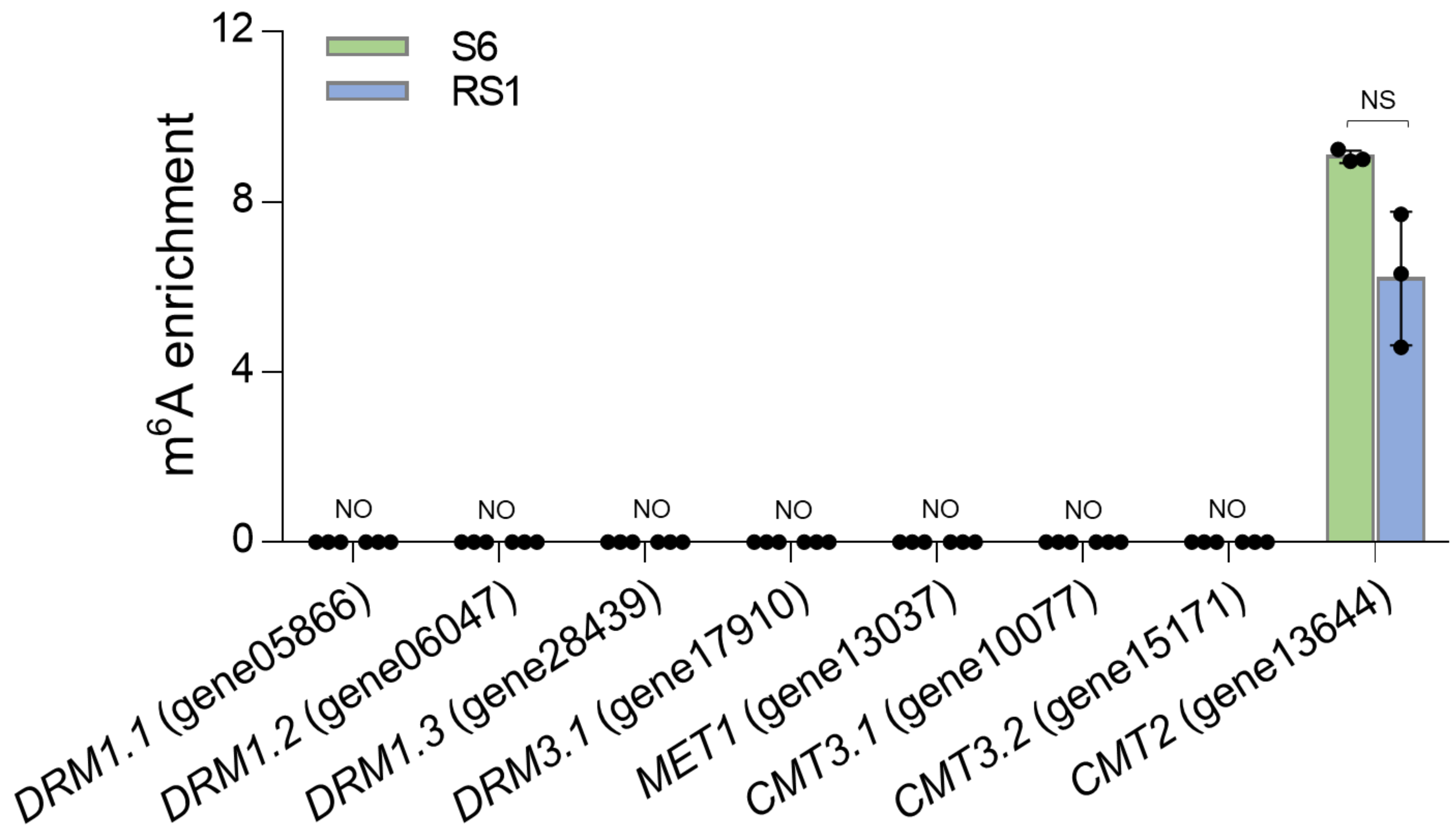

Additional file 1: Figure S16. m^{6}A enrichment for DNA methyltransferase genes from m^{6}A-seq data. Data are presented as mean ± standard deviation (n = 3). 'NO' represents no confident m^{6}A peaks identified by m^{6}A-seq. NS, no significance.

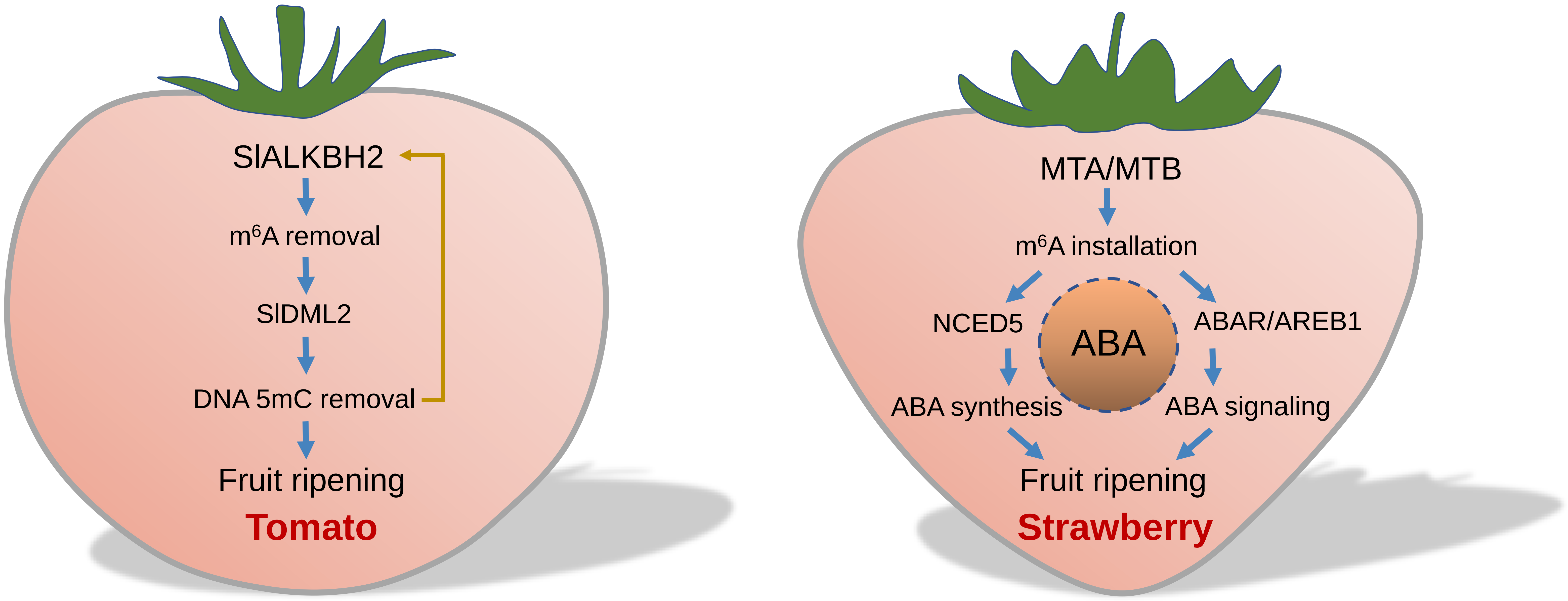

Additional file 1: Figure S17. Regulatory model for the m^{6}A-mediated ripening in climacteric tomato fruit and non-climacteric strawberry fruit. In the climacteric tomato fruit, SlALKBH2-mediated m^{6}A demethylation promotes mRNA stability of *SlDML2*, thereby facilitating fruit ripening. By contrast, in the non-climacteric strawberry fruit, the m^{6}A methyltransferases MTA and MTB target *NCED5*, *ABAR*, and *AREB1* in the ABA biosynthesis or signaling pathway to facilitate their mRNA stability or translation efficiency, leading to the ripening of strawberry fruit.
