# Supplementary material for: N6-methyladenosine RNA modification regulates strawberry fruit ripening in an ABA-dependent manner
Source: Genome Biol. 2021 Jun 3;22:168. doi: 10.1186/s13059-021-02385-0 (PMC8173835; doi:10.1186/s13059-021-02385-0)
Supplement: Supplementary file 25 — Additional file 25. Review history. [file 13059_2021_2385_MOESM25_ESM.docx]

Review History

**First round of review**

**Reviewer 1**

**Are you able to assess all statistics in the manuscript, including the appropriateness of statistical tests used?**

No, I do not feel adequately qualified to assess the statistics.

**Comments to author:**

This study globally identified the presence of m6A methylation in thousands of genes at three developmental stages in woodland strawberry F. vesca, and demonstrated that this modification influences either mRNA stability or translation efficiency. They found that the ABA biosynthesis and signaling genes, such as NCED5, AREB1, and ABAR, are subject to the m6A methylation during fruit ripening. Furthermore, they proved that the m6A methyltransferase genes MTA and MTB indeed regulate fruit ripening by affecting the m6A modification levels of the ABA genes. This study provided solid evidence by employing diverse approaches. More importantly, they found different regulatory mechanisms between the typical climacteric fruit tomato and typical non-climacteric fruit strawberry. Therefore, I think this is a very interesting paper with novel insights.

There are some questions that I would like to ask.

(1) Page 8, Line 31, how many genes were used for GO term enrichment analysis in Fig. 1d? More details are needed.

(2) Page 8, Line 50, how do you define TSS (transcription start site) for mRNA? Does TSS overlap with 5'-UTR? I think a number of genes don't have the 5' or 3' UTR, how do you deal with this difference?

(3) Page 11, Line 20-39, here it discussed the correlation between m6A deposition and mRNA abundance. The authors focus on the differential genes of m6A deposition, but no data on the number of differentially expressed genes. I think more careful comparison of these two data is helpful for revealing the actual effects of m6A modification. Some statistics are required to show the significance.

(4) Fig. 4g, are the full length CDS used? If so, the mutated A nucleotides seem not located in the differential m6A sites shown in Fig. 4b, at least for ABAR. Is this true? How do you choose the nucleotides to be mutated?

(5) Fig. 5c, expression of MTA is slightly upregulated from S6 to RS1. Compared with the expression level of MTA in RNAi shown in Fig. 6b, the RNAi efficiency is not very good. Is the delayed ripening an outcome of reduced MTA transcripts? Or the reduced MTA expression is due to the different ripening stages? In addition, since the transient expression assay was performed, the RNAi efficiency should be pretty variable. The error bars are very short, are they technical or biological replicates?

Page 19, Line 31, MAB should be MTB

**Reviewer 2**

**Are you able to assess all statistics in the manuscript, including the appropriateness of statistical tests used?**

No, I do not feel adequately qualified to assess the statistics.

**Comments to author:**

This study addresses m6A (m6A writer) functions in strawberry ripening. Previously the authors have reported that m6A demethylase regulates tomato fruit ripening, indicating m6A functions fruit ripening. In the manuscript, the authors profiled m6A methylone in three stages during ripening and narrowed down to three ABA-related genes for mechanism study and validate it using OE-MTA.

The authors put much efforts to show m6A methylone analysis in wild-type (figure 1-3), however, the analysis is too rough and did not analysis more deeply. Although they even performed mutation expression assay in tobacco, not in strawberry (figure 4), the m6A role and its underlying mechanism in strawberry ripening is not convinced until the authors showed siMTA knockdown phenotype in figure 6. The authors should rearrange the manuscript to show phenotype of m6A writer, m6A sequencing in both wild-type and siMTA, and then mechanism.

Comments and suggestions listed below:

1. Previously the authors have reported that m6A methylation regulates tomato fruit ripening. Thus it is not appropriate to claim that "However, it remains unclear whether mRNA m6A methylation, the epitranscriptome, is functionally conserved for ripening control" in the background.

2. Line 20-28, the authors conclude that "the possibility that new m6A peaks generate at the initiation stage of ripening" based on the more than 2 of m6A peaks were formed during ripening. However, the transcripts with one m6A peaks were decreased during ripening. Thus, the authors cannot be one-sided to conclude the results.

3. The authors did not clearly describe the results. For example, Fig 1d, GO for which genes, overlapped m6A containing genes in all three stages or just one stage? Actually, the GO showed in Fig. 1d did not mean anything. Instead, the authors showed give GO for differently m6A peaks containing genes, common m6A genes, unique genes in each stages to demonstrate that m6A plays role in strawberry ripening.

4. The key part of the manuscript is to show m6A was increased during strawberry ripening, especially at the initiation stage of ripening. The authors should put more data analysis related with differential m6A to demonstrate the conclusion, such as the m6A overlap among the three stages, the heatmap of m6A levels among the three stages, volcano plot of significant changes of m6A between RS1 vs S, RS2 vs. S1.

5. Whether was total m6A level (LC-MS/MS) changed during the ripening?

6. The authors should examine the relationship between differential m6A and gene expression. Using cumulative proportion plot and box plot analysis to whether the m6A hypermethylation/hypomethylation affect gene expression.

7. Line 34-42, the authors should perform GO analysis to reveal, not just describe.

8. Since single m6A locus detection methods for single genes (such as SCARLET or SELECT) have been developed, the authors should use such quantitative method to validate the three target genes.

9. Since the authors did not get the single base m6A position, thus they mutated multiple A sites. The authors did not clearly describe whether the chose A sites were in RRACH motifs. Whether multiple A to C/G mutations in CDS of the three genes affect the normal mRNA processing and codon translation? How could they know tobacco would methylate m6A on these m6A positions? If the specific m6A site was identified, the single base mutation would reduce the effects.

10. Fig 6 and 7, the substrate validation, the authors should used siRNA line to validate, not just OE-MTA line, because overexpression of MTA is artificial and might leak the protein to methylate other targets.

11. Since the authors already have siRNA KD lines, they should perform m6A-seq in control and siMTA KD line during S1, RS1, and RS2 stages to demonstrate the m6A functions in ripening, which might discover other important function pathways. So far, the authors put too much analysis on m6A in wild-type plants. To valid m6A functions, it is necessary to perform m6A in wild-type and writer mutant. Although the authors validated MTA and MTB interaction, however, it is not surprised to me because these two are homolog of human METTL3 and METT14, whose interaction is already confirmed in biochemistry and crystal structure.

12. The authors previously showed that m6A affects SIDML2 transcript processing, thereby affecting tomato fruit ripening. Since the gene is conserved between tomato and strawberry. Whether the homolog of SIDML2 in strawberry was also affected by m6A?

**Authors Response**

**Point-by-point responses to the reviewers’ comments:**

*We would like to thank the reviewers for their constructive and positive feedback. Based on the reviewers’ suggestions we have carried out additional experiments and substantially revised the manuscript. We believe that our manuscript has been improved by incorporating and implementing the reviewers’ comments. Details of the changes made during the revision are highlighted in the manuscript.*

Response to the comments of Reviewer #1:

This study globally identified the presence of m6A methylation in thousands of genes at three developmental stages in woodland strawberry F. vesca, and demonstrated that this modification influences either mRNA stability or translation efficiency. They found that the ABA biosynthesis and signaling genes, such as NCED5, AREB1, and ABAR, are subject to the m6A methylation during fruit ripening. Furthermore, they proved that the m6A methyltransferase genes MTA and MTB indeed regulate fruit ripening by affecting the m6A modification levels of the ABA genes. This study provided solid evidence by employing diverse approaches. More importantly, they found different regulatory mechanisms between the typical climacteric fruit tomato and typical non-climacteric fruit strawberry. Therefore, I think this is a very interesting paper with novel insights.

Question 1: Page 8, Line 31, how many genes were used for GO term enrichment analysis in Fig. 1d? More details are needed.

*Response: Fig. 1d shows the GO enrichment for all of the 10072 m6A-modified genes identified in strawberry fruit at three different stages. We have realized that this GO analysis appears to be rough. Therefore, we reanalyzed the GO term enrichment, using genes containing differential, non-differential, and ripening-specific m6A peaks. The results are now shown in Additional file 1: Figure S7 and described by the following sentences (page 11, line 6-39).*

*“To explore the biological significance of genes with dynamic m6A modification, we performed Gene Ontology (GO) analysis on genes containing differential, non-differential, and ripening-specific m6A peaks. In line with the progression of fruit development and ripening, genes harboring ripening-specific m6A peaks were mostly annotated to developmental pathways, including response to hormone stimulus, developmental process, histone modification, small molecular biosynthetic process, and protein transport (Additional file 1: Figure S7a). Similar enrichment was observed for genes covering differential m6A peaks at the initiation stage of ripening (from S6 to RS1) (Additional file 1: Figure S7b). In contrast, genes with non-differential m6A peaks were enriched in multiple biological processes in addition to developmental pathways (Additional file 1: Figure S7c). These results suggest that dynamic changes in m6A modification are responsible for those genes to exert their functions during fruit development and ripening.”*

Question 2: Page 8, Line 50, how do you define TSS (transcription start site) for mRNA? Does TSS overlap with 5'-UTR? I think a number of genes don't have the 5' or 3' UTR, how do you deal with this difference?

*Response: We thank the reviewer for pointing this out. In our study, the nucleotide sites in mRNAs complementary with TSS (transcription start site) in DNA sequences were defined as the TSS for mRNA. The TSS region does not overlap with 5’ UTR region when we divide the transcript segments, as described previously (Nature, 2014, 505:117-120). To avoid misunderstanding, we have now incorporated m6A peaks within TSS into those within 5’ UTR. Fig. 2 and 3 and the corresponding contents have been revised.*

*The m6A-modified genes were identified by reads mapping and peaks annotation, and only genes with m6A peaks in their mRNA segments were regarded as m6A-modified genes. Therefore, for m6A-targeted genes without 5’ or 3’ UTR, the m6A modification should be located in CDS or stop codon region.*

Question 3: Page 11, Line 20-39, here it discussed the correlation between m6A deposition and mRNA abundance. The authors focus on the differential genes of m6A deposition, but no data on the number of differentially expressed genes. I think more careful comparison of these two data is helpful for revealing the actual effects of m6A modification. Some statistics are required to show the significance.

*Response: The number of differentially expressed genes has now been described on page 11, line 53-56. According to your suggestion, we have carried out additional analyses, using cumulative proportion plot and box plot analysis to explore the relationship between m6A deposition and mRNA abundance. The results are now shown in Fig. 3g-h and described by the following sentences.*

*Page 11, 53-59; Page 12, 1-6: “There were 3562 upregulated and 3583 downregulated genes, respectively, in RS1 compared to S6 fruits. Comparison of these differentially expressed genes with the transcripts containing differential m6A modification indicated that among the 1398 transcripts with hypermethylated m6A peaks in fruit at RS1 compared to those at S6, 440 and 147 transcripts displayed higher and lower expression levels, respectively (Fig. 3f; Additional file 2: Table S13).”*

*Page 12, line 15-39: “We subsequently extended this analysis to the whole transcriptome with all m6A-modified genes. Indeed, genes bearing hypermethylated or hypomethylated m6A peaks in fruit at RS1 compared to S6 predominantly exhibited higher expression (Fig. 3g, h). This trend did not exist for non-differential m6A-modified genes (Fig. 3g, h). Considering the distribution characteristics of hypermethylated m6A (highly enriched in the CDS region) and hypomethylated m6A (mainly distributed around the stop codon or within the 3’ UTR) (Fig. 3e), it was proposed that m6A deposition in CDS region overall exhibits a positive effect on mRNA abundance, while m6A modification around the stop codon or within the 3’ UTR is generally negatively correlated with the abundance of the transcripts.”*

*Page 12, line 45-53: “The negative role of m6A modification on mRNA abundance was also observed in transcripts with differential m6A peaks identified after ripening initiation (Fig. 3g, h; Additional file 2: Table S15, S16), which were mainly distributed around the stop codon or within the 3’ UTR (Fig. 3e).”*

Question 4: Fig. 4g, are the full length CDS used? If so, the mutated A nucleotides seem not located in the differential m6A sites shown in Fig. 4b, at least for ABAR. Is this true? How do you choose the nucleotides to be mutated?

*Response: The full-length CDS was used for the experiments. The schematic diagrams have been adjusted to correctly reflect the location of the differential m6A sites shown in Fig. 4b. The nucleotides to be mutated were chosen based on our m6A-seq analysis, which identified the differential m6A peaks containing the RRACH consensus sequence. To validate the putative m6A sites identified in the m6A-seq, we have carried out additional experiments using SELECT analysis (Additional file1: Figure S9), a method for detection of single m6A locus (Angew Chem Int Ed Engl, 2018, 57:15995-16000). Fig. 4g-k has been revised according to the results of SELECT.*

Question 5: Fig. 5c, expression of MTA is slightly upregulated from S6 to RS1. Compared with the expression level of MTA in RNAi shown in Fig. 6b, the RNAi efficiency is not very good. Is the delayed ripening an outcome of reduced MTA transcripts? Or the reduced MTA expression is due to the different ripening stages? In addition, since the transient expression assay was performed, the RNAi efficiency should be pretty variable. The error bars are very short, are they technical or biological replicates?

*Response: We thank the reviewer for this comment. The RNAi experiment was conducted more than three biological replicates and the phenotype of delayed ripening can be nicely repeated. A representative result, in which the error bars are from technique replicates, was presented, which may lead to the short of error bars. To avoid misunderstanding, a sentence “The experiments were performed with more than three highly reproducible biological replicates and the representative results are presented.” has been added in the figure legends (page 61, line 11-15).*

*In our study, we found that fruits of the control display a visible change in color at the seventh days after agroinfiltration, while fruits of the RNAi maintain green at this stage. The repression of MTA expression was repeatedly observed from the fourth days, when the RNAi fruits displayed the same ripening stage as the control, to the seventh days after transient transformation. These results indicated that the delayed ripening was caused by the repression of MTA. Actually, previous studies of MTA or MTB function in Arabidopsis revealed that a 30%-50% decrease in MTA or MTB mRNA levels can cause dramatical phenotype of seedling growth, implicating the biological function and significance of these two m6A methyltransferases (New phytol, 2017, 215:157-172; Plant J, 2021, doi:10.1111/TPJ.15270).*

Question 6: Page 19, Line 31, MAB should be MTB

*Response: “MAB” has been revised to “MTB”.*

Response to the comments of Reviewer #2:

This study addresses m6A (m6A writer) functions in strawberry ripening. Previously the authors have reported that m6A demethylase regulates tomato fruit ripening, indicating m6A functions fruit ripening. In the manuscript, the authors profiled m6A methylone in three stages during ripening and narrowed down to three ABA-related genes for mechanism study and validate it using OE-MTA.

The authors put much efforts to show m6A methylone analysis in wild-type (figure 1-3), however, the analysis is too rough and did not analysis more deeply. Although they even performed mutation expression assay in tobacco, not in strawberry (figure 4), the m6A role and its underlying mechanism in strawberry ripening is not convinced until the authors showed siMTA knockdown phenotype in figure 6. The authors should rearrange the manuscript to show phenotype of m6A writer, m6A sequencing in both wild-type and siMTA, and then mechanism.

Question 1: Previously the authors have reported that m6A methylation regulates tomato fruit ripening. Thus it is not appropriate to claim that "However, it remains unclear whether mRNA m6A methylation, the epitranscriptome, is functionally conserved for ripening control" in the background.

*Response: We thank the reviewer for this comment. The regulatory mechanisms for fruit ripening are quite different between climacteric and non-climacteric fruit. Although m6A methylation was shown to regulate ripening of tomato, a typical climacteric fruit, it is not clear whether m6A is evolutionarily conserved among different types of fruits and responsible for the regulation of ripening in non-climacteric fruit. In this study, we sought to investigate whether similar mechanisms of ripening regulation by m6A exist in non-climacteric fruit. To avoid misunderstanding, the sentence has been revised to “However, it remains unclear whether mRNA m6A methylation, the epitranscriptome, is functionally conserved for ripening control among different types of fruits.”.*

Question 2: Line 20-28, the authors conclude that "the possibility that new m6A peaks generate at the initiation stage of ripening" based on the more than 2 of m6A peaks were formed during ripening. However, the transcripts with one m6A peaks were decreased during ripening. Thus, the authors cannot be one-sided to conclude the results.

*Response: According to suggestion, “raising the possibility that new m6A peaks generate at the initiation stage of ripening.” has been deleted.*

Question 3: The authors did not clearly describe the results. For example, Fig 1d, GO for which genes, overlapped m6A containing genes in all three stages or just one stage? Actually, the GO showed in Fig. 1d did not mean anything. Instead, the authors should give GO for differently m6A peaks containing genes, common m6A genes, unique genes in each stages to demonstrate that m6A plays role in strawberry ripening.

*Response: Thanks for your comment. Fig. 1d shows the GO enrichment for all of the 10072 m6A-modified genes identified in strawberry fruit at three different stages. According to your suggestion, we have reanalyzed the GO term enrichment on genes containing differential, non-differential, and ripening-specific m6A peaks. The results are now shown in Additional file 1: Figure S7 and described by the following sentences (page 11, line 6-39).*

*“To explore the biological significance of genes with dynamic m6A modification, we performed Gene Ontology (GO) analysis on genes containing differential, non-differential, and ripening-specific m6A peaks. In line with the progression of fruit development and ripening, genes harboring ripening-specific m6A peaks were mostly annotated to developmental pathways, including response to hormone stimulus, developmental process, histone modification, small molecular biosynthetic process, and protein transport (Additional file 1: Figure S7a). Similar enrichment was observed for genes covering differential m6A peaks at the initiation stage of ripening (from S6 to RS1) (Additional file 1: Figure S7b). In contrast, genes with non-differential m6A peaks were enriched in multiple biological processes in addition to developmental pathways (Additional file 1: Figure S7c). These results suggest that dynamic changes in m6A modification are responsible for those genes to exert their functions during fruit development and ripening.”*

Question 4: The key part of the manuscript is to show m6A was increased during strawberry ripening, especially at the initiation stage of ripening. The authors should put more data analysis related with differential m6A to demonstrate the conclusion, such as the m6A overlap among the three stages, the heatmap of m6A levels among the three stages, volcano plot of significant changes of m6A between RS1 vs S6, RS2 vs. RS1.

*Response: According to your suggestion, we have carried out more data analyses related with differential m6A, including volcano plots and heat maps of differential m6A peaks between RS1 vs. S6 and RS2 vs. RS1. The results are now presented in Fig. 3a-d.*

*In this study, we identified a total of 1608 hypermethylated m6A peaks which were highly enriched in the CDS region and 865 hypomethylated m6A peaks which were mainly distributed around the stop codon or within the 3’ UTR at the initiation stage of ripening (from S6 to RS1). By contrast, only 113 hypermethylated m6A peaks and 102 hypomethylated m6A peaks, all highly enriched around the stop codon or within the 3’ UTR, were identified in fruit at RS3 compared to those at RS1. We did not observe an apparent increase in total m6A levels during strawberry ripening as revealed by LC-MS/MS, which might be caused by the different m6A enrichment values between hypermethylated and hypomethylated peaks (Additional file 2: Table S6, S7).*

Question 5: Whether was total m6A level (LC-MS/MS) changed during the ripening?

*Response: We have detected the total m6A levels using LC-MS/MS and found that there were no significant changes in total m6A level during strawberry fruit ripening. Instead, a dramatic change in the topology of m6A methylome (Fig. 2) and an extensive hypermethylation (mainly in the CDS region) and hypermethylation (mainly around the stop codon or within the 3’ UTR) (Fig. 3) at the initiation stage of ripening were observed. This might be caused by the different m6A enrichment values between hypermethylated and hypomethylated peaks (Additional file 2: Table S6, S7). The results of total m6A levels are now shown in Additional file 1: Figure S5 and described on page 10, line 31-39 as follows.*

*“We did not observe an apparent increase in total m6A levels during strawberry ripening as revealed by LC-MS/MS (Additional file 1: Figure S5), which might be caused by the different m6A enrichment values between hypermethylated and hypomethylated peaks (Additional file 2: Table S6, S7).”*

Question 6: The authors should examine the relationship between differential m6A and gene expression. Using cumulative proportion plot and box plot analysis to whether the m6A hypermethylation/hypomethylation affect gene expression.

*Response: According to your suggestion, we have examined the relationship between differential m6A and gene expression using cumulative proportion plot and box plot analysis. The results are now presented in Fig. 3g-h and described by the following sentences.*

*Page 12, line 14-39: “We subsequently extended this analysis to the whole transcriptome with all m6A-modified genes. Indeed, genes bearing hypermethylated or hypomethylated m6A peaks in fruit at RS1 compared to S6 predominantly exhibited higher expression (Fig. 3g, h). This trend did not exist for non-differential m6A-modified genes (Fig. 3g, h). Considering the distribution characteristics of hypermethylated m6A (highly enriched in the CDS region) and hypomethylated m6A (mainly distributed around the stop codon or within the 3’ UTR) (Fig. 3e), it was proposed that m6A deposition in CDS region overall exhibits a positive effect on mRNA abundance, while m6A modification around the stop codon or within the 3’ UTR is generally negatively correlated with the abundance of the transcripts.”*

*Page 12, line 45-53: “The negative role of m6A modification on mRNA abundance was also observed in transcripts with differential m6A peaks identified after ripening initiation (Fig. 3g, h; Additional file 2: Table S15, S16), which were mainly distributed around the stop codon or within the 3’ UTR (Fig. 3e).”*

Question 7: Line 34-42, the authors should perform GO analysis to reveal, not just describe.

*Response: Thanks for your suggestion. We have performed GO analysis on ripening-induced and ripening-repressed transcripts that contain differential m6A modification during the ripening. The results are now shown in Additional file 1: Figure S7d and described on page 13, line 1-12 as follows.*

*“GO analysis revealed that these genes were enriched in processes such as multicellular organismal development, developmental process, nucleocytoplasmic transport, and anatomical structure development (Additional file 1: Figure S7d), implicating the involvement of m6A methylation in the regulation of strawberry fruit ripening.”*

Question 8: Since single m6A locus detection methods for single genes (such as SCARLET or SELECT) have been developed, the authors should use such quantitative method to validate the three target genes.

*Response: We have carried out additional experiments using SELECT to validate the three target genes. The results indicated that all the three genes contain at least one exact m6A site, which belongs to the putative m6A sites that we previously mutated and analyzed in the tobacco system. The results are now shown in Additional file1: Figure S9 and described on page 14, line 20-31 as follows.*

*“The intact CDS sequences of the three genes and their mutated forms in which the specific m6A sites identified in m6A-seq and validated by SELECT analysis (Additional file 1: Figure S9), a method for detection of single m6A locus [49], were mutated from A to guanosine (G) were separately inserted into the vector for transient expression in the Nicotiana benthamiana leaves (Fig. 4g).”*

Question 9: Since the authors did not get the single base m6A position, thus they mutated multiple A sites. The authors did not clearly describe whether the chose A sites were in RRACH motifs. Whether multiple A to C/G mutations in CDS of the three genes affect the normal mRNA processing and codon translation? How could they know tobacco would methylate m6A on these m6A positions? If the specific m6A site was identified, the single base mutation would reduce the effects.

*Response: We thank the reviewer for this comment. We identified specific m6A sites (A145 in NCED5, A133 in ABAR, and A1018 in AREB1) in strawberry fruit by using the SELECT analysis (Additional file1: Figure S9a). These m6A sites belong to the putative m6A sites located in the RRACH motifs that we previously chose for mutation analysis. The specific m6A sites could be identified in tobacco leaves by SELECT (Additional file1: Figure S9b), indicating that the tobacco system is suitable for m6A methylation analysis of the target genes. To avoid multiple effects that might occur in our original analysis, the experiments have been redone by mutation of single base m6A site that were identified by SELECT. Similar results are observed, which are now shown in Fig. 4g-k.*

Question 10: Fig 6 and 7, the substrate validation, the authors should use siRNA line to validate, not just OE-MTA line, because overexpression of MTA is artificial and might leak the protein to methylate other targets.

*Response: According to your suggestion, the siRNA-MTA line has been used to perform m6A-IP-PCR, RT-qPCR, mRNA stability, and translation efficiency assay for the substrate validation. The data confirm that MTA indeed targets the genes in the ABA pathway and regulates the mRNA stability or translation efficiency of these genes. These results are now described on page 17-18 and shown in Fig. 6 and 7.*

Question 11: Since the authors already have siRNA KD lines, they should perform m6A-seq in control and siMTA KD line during S1, RS1, and RS2 stages to demonstrate the m6A functions in ripening, which might discover other important function pathways. So far, the authors put too much analysis on m6A in wild-type plants. To valid m6A functions, it is necessary to perform m6A in wild-type and writer mutant. Although the authors validated MTA and MTB interaction, however, it is not surprised to me because these two are homolog of human METTL3 and METT14, whose interaction is already confirmed in biochemistry and crystal structure.

*Response: We thank the reviewer for pointing this out. According to your comments, we have performed m6A-seq on fruits from the MTA RNAi and the control. A total of 8693 and 8704 confident m6A peaks within 7933 and 5180 gene transcripts in the MTA RNAi fruits and the control, respectively, were identified. Compared with the control, we identified 2520 hypomethylated m6A peaks and 1510 hypermethylated m6A peaks in the MTA RNAi fruits, indicating an overall m6A hypomethylation when the MTA was silenced. The hypomethylated m6A peaks were mainly distributed in the CDS region, while the hypermethylated m6A peaks were highly enriched around the stop codon or within the 3’ UTR. This is similar to the distribution of differential m6A peaks identified during fruit ripening. By analyzing the overall expression of m6A-modified transcripts based on our RNA-seq data, we found that genes harboring hypomethylated m6A modification in the MTA RNAi fruits were more likely downregulated, suggesting a positive correlation between MTA-mediated m6A methylation and mRNA abundance. GO analysis revealed that genes containing hypomethylated m6A modification were significantly annotated to biological processes including response to hormone stimulus, developmental process, multicellular organismal development, cell wall organization, and secondary metabolic process, which are prerequisites for fruit development and ripening. We found that a number of ripening-related genes, including those involved in anthocyanin biosynthesis, sugar metabolism, cell wall organization and degradation, and hormone signaling, displayed hypomethylation in the MTA RNAi fruits. Together, these results demonstrated that MTA is indispensable for global m6A mRNA methylation, which ensures the proper progression of fruit ripening of strawberry by targeting ripening-related genes among various biological processes. These results are now shown in Fig. 8 and described on page 19-21.*

Question 12: The authors previously showed that m6A affects SIDML2 transcript processing, thereby affecting tomato fruit ripening. Since the gene is conserved between tomato and strawberry. Whether the homolog of SIDML2 in strawberry was also affected by m6A?

*Response: Thanks for your comment. There are four SlDML2 homologs, FveDME1, FveROS1.1, FveROS1.2, and FveROS1.3, in the strawberry genome. Our m6A-seq analysis revealed that FveDME1 and FveROS1.1 contains differential m6A peaks at the onset of fruit ripening, while FveROS1.2 and FveROS1.3 are not m6A-modified. However, none of these genes showed significantly increased expression during fruit ripening, suggesting that SlDML2 homologs might be dispensable for m6A-mediated regulation of ripening in strawberry fruit. Our results are consistent with previous study (Genome Biol, 2018, 19:212), showing that the ripening-related DNA hypomethylation in strawberry is governed by components in the RNA-directed DNA methylation (RdDM) pathway rather than those in the demethylation pathway. This has been discussed on page 22, line 22-59 and the results are shown in Additional file 1: Figure S15.*

**Second round of review**

**Reviewer 1**

I think the authors have answered all my questions. The manuscript is largely improved and acceptable for publication.

**Reviewer 2**

The authors have addressed my questions.
